# A Phenomenological Model for the Photocurrent Transient Relaxation Observed in ZnO-Based Photodetector Devices

**DOI:** 10.3390/s130809921

**Published:** 2013-08-05

**Authors:** James C. Moore, Cody V. Thompson

**Affiliations:** Department of Chemistry and Physics, Coastal Carolina University, Conway, SC 29528, USA E-Mail: cvthomps@g.coastal.edu

**Keywords:** zinc oxide, ZnO, ultraviolet, photodetector, persistent photoconductivity, photoresponse

## Abstract

We present a phenomenological model for the photocurrent transient relaxation observed in ZnO-based metal-semiconductor-metal (MSM) planar photodetector devices based on time-resolved surface band bending. Surface band bending decreases during illumination, due to migration of photogenerated holes to the surface. Immediately after turning off illumination, conduction-band electrons must overcome a relatively low energy barrier to recombine with photogenerated holes at the surface; however, with increasing time, the adsorption of oxygen at the surface or electron trapping in the depletion region increases band bending, resulting in an increased bulk/surface energy barrier that slows the transport of photogenerated electrons. We present a complex rate equation based on thermionic transition of charge carriers to and from the surface and numerically fit this model to transient photocurrent measurements of several MSM planar ZnO photodetectors at variable temperature. Fitting parameters are found to be consistent with measured values in the literature. An understanding of the mechanism for persistent photoconductivity could lead to mitigation in future device applications.

## 1. Introduction

Bulk and thin-film ZnO materials have attracted a great deal of attention, primarily due to their potential applications as photodetectors, gas sensors and as electronic and light-emitting nano-devices. Zinc oxide-based devices have the potential to outperform other wide band gap materials and, in the future, may replace nitride-based semiconductors [1]. Specifically, ZnO has attracted a great deal of attention for its ultraviolet (UV) light detection properties, with several studies concerning photoconductivity [2,3]. Applications for UV photodetectors include air-quality monitoring, missile warning systems and gas detection [4–7].

Photodetectors are typically fabricated from ZnO nanowires and thin films using such techniques as the relatively costly chemical vapor deposition processes [8,9]. However, lower-cost methods, such as radio frequency and direct current (dc) magnetron sputtering, have been employed. Recently, a technique for low-cost growth has been developed, where Zinc-metal films are grown and, then, thermally oxidized to produce polycrystalline ZnO films [2,10]. Highly resistive films with polycrystalline structure have resulted when starting with approximately 200 nm-thick Zn-metallic films [11–13]. These ZnO films have been utilized as a base for ZnO ultraviolet photodetectors, with various metals applied as contacts for photodetectors having a metal-semiconductor-metal (MSM) planar structure [14]. Specifically, Zheng *et al.* fabricated ZnO photodetectors on glass substrates with Al contacts, which showed a large UV photoresponse [15]. Ali *et al.* report ultraviolet photodetectors with high gain grown via thermal oxidation with Pd Schottky contacts [16]. In the present study, we have used a similar process to fabricate ZnO photodetectors on sapphire substrates with ohmic Al contacts.

A persistence in the photoconductivity post-illumination has been reported for ZnO-based UV photodetectors, and we see similar phenomena in our fabricated devices [2,17,18]. This phenomenon occurs when electromagnetic radiation is removed from exposed photoconductors after the point of photocurrent saturation. Rather than immediately returning to a base level of photocurrent, typical relaxation times for decreasing photocurrent are observed to range from hours to several days. Often, persistent photoconductivity is attributed to the rates of various excitation-recombination mechanisms. A two-step photocurrent relaxation, consisting of an initial sharp drop in photoconductivity, followed by a slow decline, appears throughout previous literature [17,19–21].

Addressing the problem of persistent photoconductivity has become a priority in electronic materials research, since solving the problem could lead to more reliable and faster response photodetectors. A variety of schemes to eliminate persistent photoconductivity have been developed, where enhancement of the photogain can occur simultaneously with reduction in photoresponse/recovery times. Specifically, the literature discusses the introduction of Schottky junctions, surface modifications, the reduction of nanowire sizes and the introduction of core-shell geometry and network structures [22–26].

There is significant debate in the literature about the underlying processes governing the persistent photoconductivity observed for ZnO [17]. The engineering, device and materials literature has relied on a non-physical fitting function for the time-dependent transient behavior to make comparisons between materials, growth parameters and systems [17,27,28]. In this article, we discuss a phenomenological model for the persistent photoconductivity observed in ZnO photodetector devices based on time-resolved surface band bending and electron transport at the interface. We have numerically fit a complex rate equation to transient photocurrent measurements of several MSM planar ZnO photodetectors at variable temperature, with fitting parameters found to correspond to directly measured values in the literature. An understanding of the mechanism for persistent photoconductivity could lead to mitigation in future device applications.

This article is organized into four main sections: a discussion of the fabrication and characterization of the ZnO photodetectors under study (Section 2), the theoretical background and development of the phenomenological model (Section 3), experimental photocurrent results and comparison to the model (Section 4) and a discussion of the implications and limitations of the model as applied to empirical photocurrent relaxation (Section 5).

## 2. Fabrication and Characterization

Zinc oxide MSM photodetectors with Al contacts were fabricated via sputter deposition, with ZnO thin films on *c*-plane sapphire grown using a thermal oxidation method described in detail elsewhere [2,29]. Several other groups report successful growth of ZnO thin films via thermal oxidation. Cho *et al.* demonstrate growth of un-doped ZnO films with a polycrystalline wurtzite structure, where grain size is seen to increase with annealing temperature [10]. Wang *et al.* report weak deep level photoluminescence (PL) emission bands compared to UV emission and green band intensity that increases at higher temperatures when films are oxidized in air [11]. However, Chen *et al.* show decreasing deep level band intensities with increasing temperature when films are oxidized in ambient oxygen via a two-step process [12]. Li *et al.* used a direct-current magnetron sputtering system to deposit Zn on glass substrates in Ar and a combination of Ar and O_2_ [13]. It was discovered that the films deposited in Ar had a large particle size and strong UV emission. An addition of O_2_ to the Ar led to a porous structure with high optical transmittance, but decreased photoluminescence intensity. Y.I. Alivov *et al.* found that the optical properties of ZnO films improve with an increase in annealing temperature and, to an extent, with a decrease in oxygen partial pressure [30]. All groups demonstrate highly resistive films with polycrystalline structure when starting with approximately 200 nm-thick Zn-metallic films.

In this section, we discuss the growth and characterization of ZnO thin films via thermal oxidation of metallic Zn (Section 2.1) and the fabrication and *IV* characterization of MSM photodetectors on these base films (Section 2.2).

### 2.1. Film Growth and Characterization

For the present study, Zinc films were deposited on *c*-plane sapphire substrates via direct current sputter deposition. Sapphire substrates where chosen because of their UV transparency and thermal properties conducive to high temperature annealing. Metallic Zn targets were obtained commercially and had a purity of 99.99%. Before deposition, substrates were cleaned via immersion in acetone, ultrasonically cleaned in methanol and rinsed in deionized water. Chamber base pressure was maintained between 1.0 × 10^−5^ and 2.5 × 10^−5^ mbars. A gate valve between the chamber and the pump was utilized as a throttle to maintain an Ar pressure of approximately 2 × 10^−5^ mbars. Sputtering power was maintained between 10–20 W, with the substrate located approximately 10 cm from the sputter source. Deposition times ranged from 15–40 min, resulting in film thicknesses between 100 nm and 400 nm, as measured via an atomic force microscopy (AFM) profile and reflectometry. Thermal oxidation of the Zn metal films was carried out in an air-ambient muffle furnace at 573 K for 9–24 h to insure complete oxidation. Some films were then re-annealed for 1 h at 873, 1,173 and 1,473 K. The resulting ZnO films where between 200–600 nm in thickness.

Films were characterized via X-ray diffraction (XRD), atomic force microscopy (AFM) and photoluminescence (PL). The fabricated UV photodetectors were characterized via large-scale current-voltage (*IV*) measurements and transient analysis. The structural properties of the ZnO films were measured using Cu-K*α*; radiation in the range from 30° to 50°. The morphology of the films was determined via dynamic-mode AFM using an Anfatec Level AFM and approximately 300 kHz resonant aluminum backside silicon tips. Photoluminescence (PL) spectra were obtained at room temperature using a HeCd laser as an excitation source and a power P = 0.3 W/cm^2^. A picoammeter manufactured by Ix Innovations was used to measure current response, and UV illumination was provided by a deuterium lamp with power P = 2 mW/cm^2^ or a UV LED with maximum power P = 0.2 mW/cm^2^.

**Figure 1.**
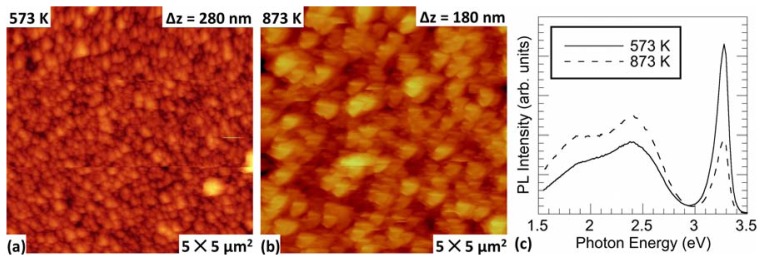
Atomic force microscopy (AFM) topography images of ZnO films fabricated via thermal oxidation of Zn metal films at an annealing temperature of (**a**) 573 K; and (**b**) 873 K; (**c**) The photoluminescence spectra of the same ZnO thin films showing yellow, green and excitonic emission at ∼2, 2.4 and 3.4 eV, respectively.

Figure 1a,b shows the surface morphology of the resulting ZnO films annealed at 573 and 873 K, respectively. The as-grown zinc film demonstrates a high surface roughness and approximately 100 nm diameter protrusions (not shown). There is no significant change in surface morphology between the annealed Zn-metal films and the oxidized films at this annealing temperature, where pre-oxidation Zn-metallic films exhibit similar morphology to that seen in Figure 1a. However, an increase in protrusion diameter and surface roughness is observed with increasing annealing temperature, as seen in Figure 1b. This change in surface morphology is consistent with other studies [10].

XRD patterns indicate that after annealing, the resulting ZnO films possess a polycrystalline hexagonal wurtzite structure without a preferred orientation (not shown). This is consistent with previous studies, where Gupta *et al.* show that the preferred orientation of ZnO thermally oxidized on glass can depend on the Zn film texture and the oxidizing agent [31]. Grain size was calculated from the Scherrer formula and XRD patterns and confirmed via AFM [32]. For films having thicknesses between 400 and 600 nm, grain size was observed to increase with an increasing annealing temperature up to 873 K, as discussed in more detail elsewhere [29]. No significant increase in grain size was observed with a continued increase in annealing temperature past 873 K. These observations are consistent with the morphological observations made using AFM and are consistent with the literature [10,16].

The photoluminescence of ZnO has been shown to include yellow, green, blue and UV emission, depending on the environments and annealing conditions in which the films were grown, and annealing temperature heavily influences PL spectra [11,12,29]. Figure 1c shows the PL spectra for the ZnO films thermally annealed at both 573 and 873 K. Spectra show asymmetric broad bands in the yellow-green region. A more narrow band in the UV is observed, which is associated with excitonic emission. It is generally accepted that the green and yellow emission results from the recombination of a delocalized electron close to the conduction band with a deeply trapped hole in the single ionized oxygen vacancy $\left(V_{0}^{+}\right)$ and the single negatively-charged interstitial oxygen ion $\left(O_{i}^{-}\right)$ centers in the bulk, respectively [33,34]. Reduction in optical quality is observed with increasing annealing temperature, which results in decreased photodetector performance. Specifically, the ratio of excitonic-to-green emission is seen to decrease, where Figure 1c shows increasing green band emission and decreasing excitonic emission with increasing temperature. This is discussed in greater detail in reference [18].

### 2.2. Fabrication of Photodetectors and Characterization

**Figure 2.**
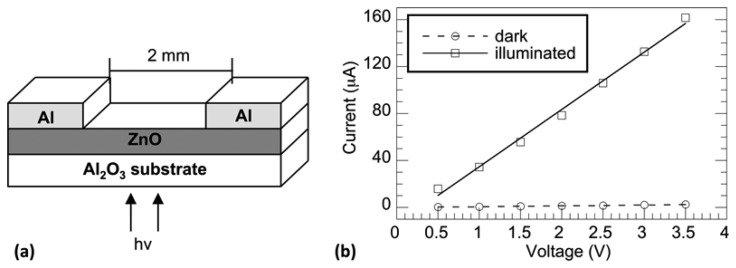
**(a)** Schematic diagram of the Al:ZnO:Al MSM planar structure; and (**b**) *IV* curves in dark and UV illumination for a photodetector annealed at 573 K. Illumination was via a deuterium lamp reactor with an intensity at the detector of 2 mW/cm^2^.

Figure 2a shows a schematic diagram of the fabricated UV photodetectors having an Al:ZnO:Al MSM planar structure. Metallic Al contacts with wide spacing (1–2 mm) were deposited on the ZnO surface via sputter deposition using a shadow masking technique. Narrow spacing (0.2–0.5 mm) contacts where deposited via a mask-less photolithography process. Aluminum targets were obtained commercially and had a purity of 99.99%. Film surfaces were cleaned similarly to substrates, as detailed above. Aluminum contacts were sputter deposited at approximately 20 W for 30 min, resulting in a contact thickness of approximately 200 nm. Photodetectors were backside illuminated. Although contact spacing and geometry did affect the photoconductivity of the fabricated devices, in this study, we are interested in the normalized photocurrent transients, which we have shown do not depend on these properties [18].

To obtain *IV* data, the samples were connected in series to a power supply and a picoammeter, and measurements were made in dark and UV illuminated conditions in ambient air. Figure 2b shows the *IV* response for both dark and illuminated conditions of a photodetector with a 573 K annealed base ZnO film. As shown, there is a significant difference in conductivity between the sample under dark conditions and the sample under UV illumination. The *IV* response for the dark condition is found to be linear, suggesting ohmic contact between the ZnO film and Al electrodes. Under illumination, however, the *IV* response is quasi-ohmic. The non-linear response seen in Figure 2b could be attributed to thermal heating of the sample during prolonged exposure to close-proximity UV illumination and/or the slow photoresponse discussed later in this manuscript. Photocurrent is determined by subtracting the illuminated current from the dark current. The negligent slope for the dark curve indicates that thermally oxidized ZnO films are highly resistive, which is consistent with reports on films grown via this method [2,29]. When illuminated via broad-spectrum visible light, the resulting rise in conductivity is observed to be much less than a factor of 10, which suggests that these detectors are quasi-visible-blind (not shown). Observed photo-induced current using below band gap visible light can be attributed to surface and defect states corresponding to the yellow and green emissions observed in the PL spectra (see Figure 1c). With increasing base-film annealing temperature, photoconductivity is seen to decrease (not shown), which is consistent with the decrease in photoemission efficiency observed in the PL spectra.

**Figure 3.**
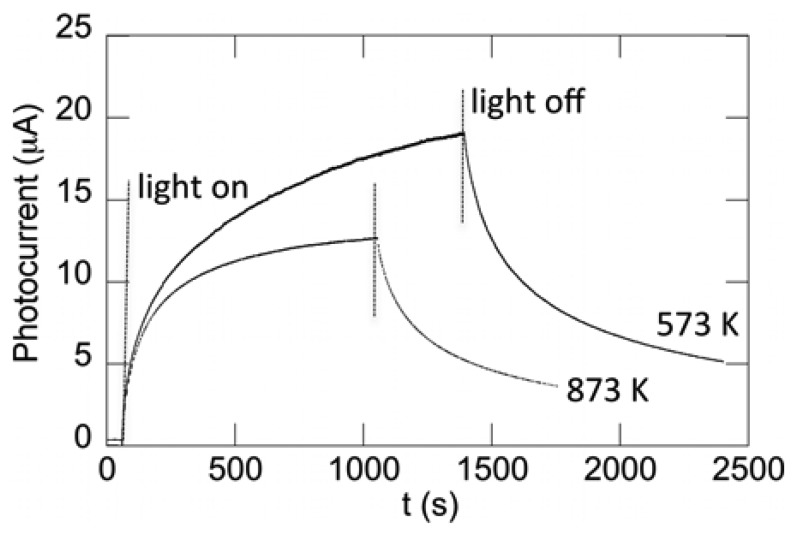
Photocurrent transients for films annealed at 573 and 873 K and held at 1 V bias. The first vertical dotted line indicates when illumination was turned on. The second vertical dotted line indicates when illumination was turned off.

Figure 3 shows the photocurrent transients for films annealed at 573 and 873 K and held at 1 V bias. The films are stored in dark and allowed to maintain at dark-condition equilibrium for up to 24 h. Subsequently, they are exposed to UV illumination, as marked by the first vertical line in the figure. Photocurrent saturation is slow, taking up to 40 min for some films. Various growth conditions and annealing temperatures affect both the level of saturation and the length of time in which the saturation point occurs. For example, Figure 3 shows that the detector based on the film annealed at 873 K reaches saturation faster, though with a lower saturation photocurrent. For both annealing temperatures, the photocurrent initially increases quickly to about 5 μA and, then, slowly approaches saturation. The rapid photocurrent rise can be explained by the initial excitation of electrons to higher energy states. The slower photocurrent rise has been attributed to photo-excited states that exhibit a short lifetime and probability for a lattice relaxation at the surface, in which deep unknown centers form when shallow donors convert into deep donors [17]. The potential energy of the lattice lowers after relaxation, which, in turn, lengthens the recapture rate. As time progresses, the surface state relaxation decreases and the relaxation time constant increases. Once saturation is achieved, illumination is removed, as indicated by the second set of vertical lines in Figure 3. A similar fast drop in photocurrent of approximately 5 μA is observed when illumination is turned off. Then, a slow process takes over to reach initial dark values, resulting in a persistence in the photoconductivity that can range from several hours to days. It is this persistent photoconductivity observed in ZnO photodetector devices that is the central topic of this study.

## 3. Phenomenological Model

There is significant debate in the literature about the underlying processes governing the persistent photoconductivity observed for ZnO. The engineering, device and materials literature has mostly relied on a non-physical fitting function for the time-dependent transient behavior to make comparisons between materials, growth parameters and systems. Several different mechanisms have been proposed, but very few have been systematically explored. In this section, we discuss Kohlrausch stretched exponential analysis (Section 3.1), and we present a phenomenological model for persistent photoconductivity that relies on surface band bending and electron transport at the surface (Section 3.2).

### 3.1. Transient Analysis Using the Kohlrausch Stretched-Exponential and Multiple-Exponential Analysis

In previous literature, the traditional approach to describe transient behavior has been to use a Kohlrausch stretched exponential function analysis, which is used to describe decays in the presence of an energy transfer in disordered systems [27,34]. The Kohlrausch function is a convenient and relatively flexible fitting function, consisting of a stretching parameter, *γ*, that distinguishes it from a classical exponential decay. The Kohlrausch function, as applied to photocurrent transient relaxation, is as follows:

(1) $$i(t)=A\exp[-{\left(t/\tau \right)}^{\gamma }]$$

where A is the saturation photocurrent, *τ* is the relaxation time constant and *γ* falls into a range between zero and one. As *γ* approaches one, the function approaches classical single-exponential behavior, and differences in energy transfer processes become indistinguishable. Kohlrausch analysis is a convenient fitting function for processes in which slight deviations from single-exponential behavior occur, due to the combination of multiple energy transfer mechanisms [27,34].

The photocurrent decay transient data obtained from our photodetectors were fitted with a Kohlrausch decay function and are discussed at length in a previous article [18]. Furthermore, a double-exponential function has been used to fit photocurrent relaxation, where the two exponential functions are used to separately analyze what is described as the “fast” and “slow” photorelaxation processes [18,35]. Although there is a wide range in time constants discussed in the literature, time constants for our detectors analyzed with both stretched and double exponentials are within the same general range (approximately 400 s for stretched exponential analysis). The significance of this broad range in reported time constants is arguable. Reemts and Kittel grew ZnO films by electrodeposition and found an order of magnitude difference in time constants within their films, which ranged between 5.30 × 10^3^ s and 2.35 × 10^4^ s [17]. Carrey *et al.* demonstrate persistent photoconductivity time constants for ZnO nanoparticles in the range of tens to thousands of seconds [27]. Studenikin *et al.* claim time constants of a range from 135 s to 1.23 × 10^4^ s for ZnO films, where they separate the decay into four exponential transients [28]. There is a variation in *γ* values, as well, with values of 0.57 and 0.40 to 0.52 in two of the experiments discussed above. In previous work, we have shown that time constants do not strongly depend on minor structural and optical properties of the films and may vary significantly, due to fabrication methods and/or the interface environment [18]. However, it is extremely difficult to ascertain the physical processes underlying the spread in reported values, since Kohlrausch analysis provides no physical mechanism, and although a double- or even quadruple-exponential allows for analysis of different speed processes, no underlying mechanism is provided. A physical phenomenological model is necessary to guide researchers and engineers in the development of new systems tailored towards decreases in photoconductivity persistence and faster device response times.

### 3.2. Photocurrent Transient Relaxation and Electron Transport at the Interface

We propose a phenomenological model that describes the transport of charge carriers from within the ZnO bulk and depletion region to its surface after the termination of illumination. Li *et al.* discuss a conceptual model, where conduction-band electrons recombine with photogenerated holes at the surface after removal of illumination [36]. Surface band bending decreases during illumination, due to migration of photogenerated holes to the surface. Immediately after turning off illumination, conduction band electrons must overcome a relatively low energy barrier to recombine with photogenerated holes at the surface; however, with increasing time, the adsorption of oxygen at the surface increases band bending, resulting in an increased bulk/surface energy barrier that slows transport of photogenerated electrons. We describe this model analytically by examining how the time-dependent band bending contributes to the bulk-surface transfer of conduction band electrons. Furthermore, net electron transfer away from the conduction band is linked to decreasing photocurrent.

**Figure 4.**
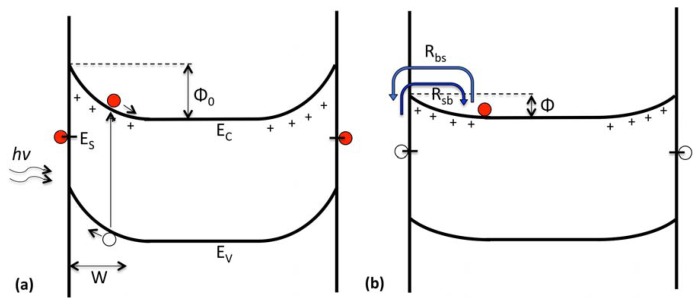
Schematic electron energy band diagram of the interface for ZnO nano-pillars showing electron transitions discussed in this study. (**a**) During the above band gap illumination, electron-hole pairs are created with photogenerated holes migrating to the surface to neutralize the native surface charge; (**b**) after the removal of illumination, electron transport from the bulk to the surface (*R_bs_*) and from the surface to the bulk (*R_sb_*) is regulated by the bulk-surface energy barriers, Φ and Φ_0_ + *E_C_* − *E_S_*, respectively

A simplified electron energy band diagram for ZnO nano-pillars on sapphire in ambient air is shown schematically in Figure 4. Negative charge at the surface having density, *n_s_*, causes upward band bending having energy, Φ. The width of the depletion region, *W*, is given by:

(2) $$W=\frac{n_{s}}{N_{D}}=\sqrt{\frac{2\Phi \epsilon \epsilon_{0}}{q^{2}N_{D}}}$$

where *ϵ*_0_ is the vacuum permittivity, *ϵ* is the dielectric constant, *q* is the electron charge and *N_D_* is the concentration of shallow donors. The band bending, Φ, is time-dependent, with Φ_0_ representing the initial, native-state band bending due to initial surface charge density, *n_s_*_0_, before illumination. During illumination, photogenerated electron-hole pairs are created, with holes migrating to the surface and electrons migrating to the bulk in the conduction band. This increase in charge carriers in the conduction band results in increased conductivity. Photogenerated holes neutralize negative surface charge, which results in a decrease in surface band bending.

As discussed by Reshchikov *et al.*, two sources of negative charge on *n*-type semiconductors have been proposed: (1) an internal mechanism from interface states, due to disorder/defects; and (2) an external mechanism, due to the adsorption of species from ambient air [37]. The exact nature of the initial negatively charged surface states is beyond the scope of this work. Also not considered is the exact mechanism for charge trapping and/or scavenging, due to the interaction with ambient air.

Thermionic transitions of electrons occur between the surface states and bulk, where such transitions obey Boltzmann statistics. In the present study, we are interested in the transient relaxation of photocurrent and will confine our analysis to the time period immediately after the termination of illumination. Low surface band bending immediately after illumination facilitates the transfer of free electrons from the bulk region, since they can easily overcome the barrier height, Φ, and become trapped at the surface. The rate of bulk-to-surface flow (*R_bs_*) is as follows:

(3) $$R_{bs}=A\exp[-\frac{\Phi (t)}{kT}]$$

where:

(4) $$A=C_{n1}N_{s}N_{C}$$

The constant, *A*, is the rate of bulk-to-surface transfer under dark conditions absent band bending. The capture of bulk electrons by surface states is described by the effective capture coefficient, *C_n_*_1_ [37,38]. The density of conduction band states is *N_c_*, and *N_s_* is the concentration of available surface states. The total amount of band bending, Φ, is time-dependent, since band bending changes as electrons are transferred. In principle, the concentration of available surface states for electrons (*N_s_*) may also be time-dependent, though we will assume it is constant for the first approximation.

Electrons that are initially trapped at the surface can overcome the potential energy barrier and migrate to the bulk (see Figure 4). Although continuous or at least several discrete surface states are present at the surface, for a first approximation, we will consider a single surface state, *E_s_*, that pins the Fermi level, *E_F_*, and controls the band bending in dark. The surface-to-bulk rate is as follows:

(5) $$R_{sb}=B(\frac{n_{C}}{n_{C0}})$$

where:

(6) $$B=C_{n2}N_{C}\exp\left[-\frac{\Phi_{0}+E_{C}-E_{F}}{kT}\right]$$

The constant, *B*, is the rate of change for the density of charge carriers moving into the conduction band immediately after removal of illumination. The capture of surface electrons by the bulk is described by the effective capture coefficient, *C_n2_* [37,38]. The native band bending is Φ_0_, and *E_C_* is the conduction band edge energy. The time-dependent density of photogenerated negatively charged states in the conduction band is *n_C_*, and the initial density immediately after the termination of illumination is *n_C0_*. This notation should not be confused with *n_s0_*, which is the density of charged surface states in dark after a very long time.

Photogenerated electrons in the conduction band may also recombine with photogenerated holes present within the valence band that have not migrated to the surface. The rate will depend on the photoemission efficiency, *α_eff_*, and the density of valance band charge carriers, *n_V_*. The rate of band-to-band transitions, *R_bb_*, is as follows:

(7) $$R_{bb}=\alpha_{\text{eff}}(n_{V})$$

where the density of available holes in the valance band is equal to the density of photogenerated conduction band electrons minus the density of holes that have migrated to the surface/grain boundary. For small enough nanowire diameter or grain size, and assuming a sufficient number of surface traps, then nearly all photogenerated holes will migrate, resulting in *n_V_* ≈ 0. It should be pointed out that this approximation is appropriate for materials exhibiting a small ratio of photogenerated charge density to native surface charge. For *n_C_*/*n_s_*_0_ > 1, no initial band bending would be present, and therefore, excess photogenerated holes could remain in the valence band at *t* = 0; however, this transition is expected to be extremely fast relative to other processes and results in steady-state photoluminescence during illumination, contributing little to the photocurrent.

We are interested in the rate of conduction band electron transfer, since the photogenerated conduction band charge carriers are the main contributor to the photocurrent. The rate of surface-to-bulk transfer is strongly dependent on the time-dependent band bending, Φ, which, in turn, depends on the density of charged surface states, *n_s_*. From Equation (2), we find:

(8) $$\Phi =\Phi_{0}{\left(\frac{n_{s}}{n_{s0}}\right)}^{2}$$

The density of negative charge at the surface, *n_s_*, is equal to the native charged surface states in dark, *n_s0_*, minus the density of photogenerated holes that migrate to the surface:

(9) $$n_{s}=n_{s0}-n_{C}$$

where we again assume small enough nanowire diameter, grain size or film thickness and a sufficient number of surface traps that nearly all photogenerated holes will migrate, such that the density is equal to the density of photogenerated negative charge in the conduction band, *n_C_*.

The rate equation describing the normalized transfer of charge from the conduction band to the surface, and from the surface to the conduction band, is determined by combining Equations (3), (5), (8) and (9):

(10) $$\frac{d}{dt}[\frac{n_{C}}{n_{C0}}]=-A\exp[-\frac{\Phi_{0}}{\eta kT}{\left(1-\beta \frac{n_{C}}{n_{C0}}\right)}^{2}]+B(\frac{n_{C}}{n_{C0}})$$

where we have introduced a phenomenological factor, *η* for generality similar to the ideality factor in Schottky diodes [39]. Kronik and Shapira discuss the variation of *η* from 0.5 to two for surface photovoltage phenomena, where *η* < 1 corresponds to charge trapping at the surface due to illumination and *η* ≈ 2 corresponds to trapping in the depletion region [40]. We also define the ratio, *β* = *n_C0_*/*n_s_*_0_, which is the ratio of the total density of photogenerated electron/hole pairs to the density of surface charge available for hole migration. This valu should depend on the illumination intensity and temperature.

In Section 4, we present normalized photocurrent data as a function of time. The normalized photocurrent through a semiconductor can be approximated via the Drude model, where the photocurrent, *i*, is proportional to the density of photogenerated charge carriers in the conduction band, *n_C_*. Assuming a planar MSM structure and constant applied electric field, this approximation allows us to relate Equation (10) to experimentally acquired and normalized photocurrent measurements. The rate equation for the normalized photocurrent is as follows:

(11) $$\frac{d}{dt}[\frac{i}{i_{0}}]=-A\exp[-\frac{\Phi_{0}}{\eta kT}{\left(1-\beta \frac{i}{i_{0}}\right)}^{2}]+B(\frac{i}{i_{0}})$$

Equation (11) cannot be solved analytically; however, we have solved this equation numerically and are capable of fitting the rate equation to experimental photocurrent data in order to determine the coefficients and *β*. In the next section, we present data for the persistent photoconductivity observed in the devices described in Section 2.2, and we fit these data to Equation (11).

## 4. Results

In this section, we fit Equation (11) to photocurrent transient relaxation data measured for varying illumination intensity (Section 4.1), base film annealing temperature (Section 4.2) and detector operating temperature (Section 4.3). For annealing temperature measurements, we are interested in whether grain size and/or photoemission efficiency has a significant effect on relaxation rates. Furthermore, other groups report a strong dependency on illumination intensity, specifically with respect to surface photovoltage measurements [37]. Therefore, we investigate normalized photocurrent relaxation with varying illumination intensity.

### 4.1. Effect of Variation in Light Intensity

**Figure 5.**
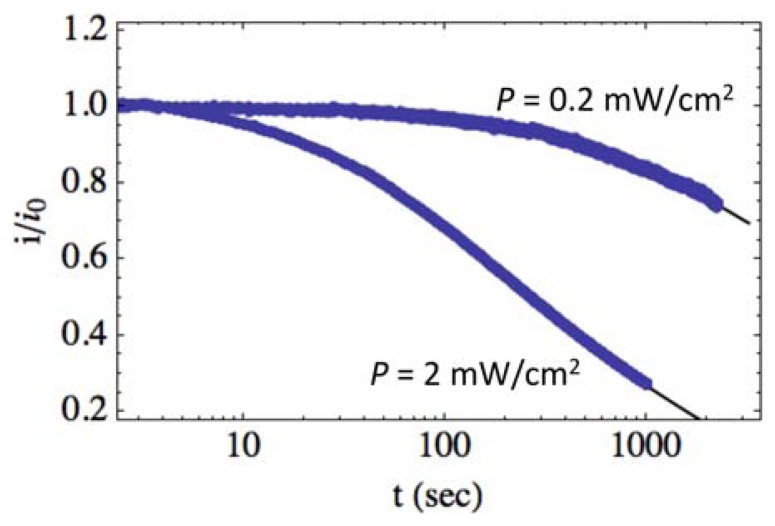
Photocurrent transient relaxation at room temperature (*T_op_* = 293 K) with increasing illumination intensity measured at *P* = 0.2 mW/cm^2^ and *P* = 2.0 mW/cm^2^ (filled circles). Experimental data is fit to Equation (11), with the best fit shown as the solid lines. For *P* = (0.2, 2.0) mW/cm^2^, coefficients of best fit are *A* = (1.5, 1.5) × 10^−2^*s*^−1^*B* = (3.1, 3.1) × 10^−4^*s*^−1^ and *β* = (0.25, 0.63). (*η* = 2, Φ_0_ = 0.3 eV). The base film was annealed at 573 K for the sample under study.

Figure 5 shows the normalized photocurrent relaxation transients for the same sample UV illuminated at *P* = 0.2 mW/cm^2^ and *P* = 2.0 mW/cm^2^ (filled circles). Greater illumination intensity results in a faster normalized relaxation, where the normalized photocurrent is seen to decrease 40% more in the same time period when intensity is increased by an order of magnitude. Equation (11) is fitted to the data shown in Figure 5 and is shown as the solid lines, with fitting parameters, *A* = (1.5, 1.5) × 10^−2^*s*^−1^, *B* = (3.1, 3.1) × 10^−4^*s*^−1^ and *β* = (0.25, 0.63) for *P* = (0.2, 2.0) mW/cm^2^. For the native band bending, we use Φ_0_ = 0.3 eV. We have also chosen *η* = 2, which corresponds to significant trapping in the depletion region and deep surface traps [17,28,40]. However, it should be pointed out that Φ_0_ and *η* could be different while maintaining the fit, so long as the ratio between the two values remains constant. This provides a range of values for Φ_0_ from approximately 0.1 to 0.3 eV for *η* from 0.5 to two. Reported values for the native ZnO surface band bending vary significantly depending on the environment and structure. Apparently, there is no band bending for a clean ZnO surface cleaved in ultrahigh vacuum [41]. In ambient air, however, chemisorption of oxygen results in band bending and electron transfer from the semiconductor to oxygen molecules or atoms, with the rate of electron transfer proportional to the oxygen pressure (10^−3^ to 20 Torr) [42]. Under illumination in vacuum, desorption of oxygen takes place, resulting in a surface photovoltage of up to 0.3 eV for single crystal ZnO [42]. Annealing causes the chemisorption of oxygen and a change from 0.2 eV of downward band bending to 0.3 eV of upward band bending [43,44]. However, very large surface band bending of ∼1.54 eV has been reported for ZnO nanowires, where the persistent photoconductivity is due to the spatial separation of photoinduced electrons and holes in the ZnO nanowire [45]. Native surface band bending for our thermally annealed films is consistent with the lower values reported for single crystal studies, even though our films demonstrate relatively large grain size (∼100–300 nm). In Section 4.3, we also determine an approximate value for the surface state energy, *E_s_*, that suggests *η* ≈ 2.

The normalized transient relaxation is found to depend only on *β*, which is physically consistent with the model. For the same photodetector operating at the same temperature, *A* and *B* should remain constant, which can be seen by examination of Equations (4) and (6). This is what is observed, as shown in Figure 5, where the fitting parameters are *A* = 1.5 × 10^−2^*s*^−1^ and *B* = 3.1 × 10^−4^*s*^−1^ for both illumination intensities. However, there is a significant difference between values for *β*. With increasing intensity, we would expect the density of photogenerated electron/hole pairs to increase, while the density of native charged surface states before illumination should remain constant. This results in an increase in *β* with increasing intensity, as observed.

### 4.2. Effect of Thermal Oxidation Annealing Temperature

**Figure 6.**
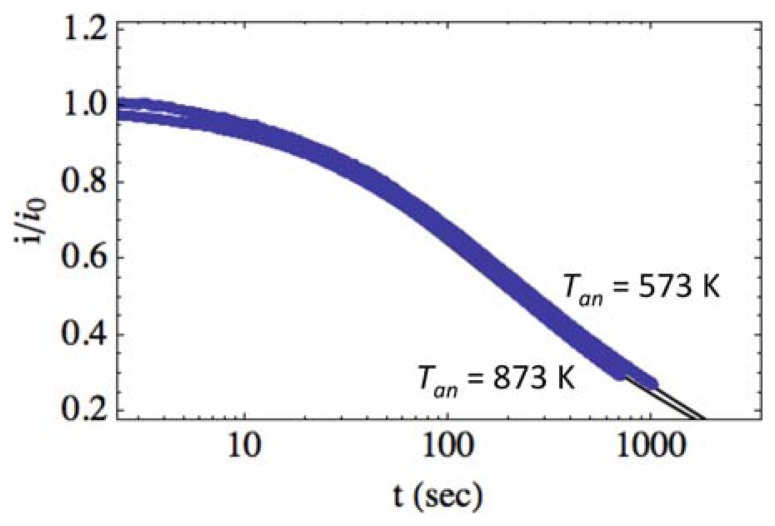
Photocurrent transient relaxation for detectors with base films annealed at *T_an_* = 573 K and *T_an_* = 873 K (filled circles) at illumination intensity *P* = 2.0 mW/cm^2^. Experimental data is fit to Equation (11), with the best fits shown as the solid lines. For *T_an_* = (573, 873) K, *A* = (1.5, 1.5) × 10^−2^*s*^−1^*B* = (3.1, 3.5) × 10^−4^*s*^−1^ and *β* = (0.63, 0.67). (*η* = 2, Φ _0_ = 0.3 eV).

Figure 6 shows the normalized photocurrent transient relaxation for photodetectors having varying base film annealing temperatures (*T_an_*, filled circles). As discussed in Section 2.1 and shown in Figure 1, grain size is seen to increase, and optical efficiency decreases with increasing annealing temperature, which is discussed in detail in previous work by our group [18,29]. To determine if these properties contribute to the transient relaxation, we fit Equation (11) to the data in Figure 6 and show the fits as solid lines. The fitting parameters were found to be *A* = (1.5, 1.5) × 10^−2^*s*^−1^, *B* = (3.1, 3.5) × 10^−4^*s*^−1^ and *β* = (0.63, 0.67) for *T_an_* = (573, 873) K, respectively. There is no significant difference between fitting parameters for the two detectors.

The similarity of the fitting parameters suggests that they are not governed by the various thermal-annealing temperatures of the films and, therefore, the grain size or the optical efficiency. This is consistent with previous literature, which suggests that the time constants are governed by the ambient environment and by the density and the depth of deep surface traps in ZnO, which affect the capture of holes and the subsequent hole emission and carrier recombination mechanisms of photocurrent decay [17,28,35]. It should be pointed out that the grain sizes for our films are relatively large and within the same order of magnitude (∼100–300 nm) and that we might expect a different result for smaller grain size or nanowire films. Furthermore, we investigate the normalized photocurrent in this study, which masks differences in the photocurrent saturation. There is a significant difference observed in photocurrent saturation between the two samples (see Figure 3). This can be attributed to the differences observed in the photoluminescence, where the low-temperature annealed film demonstrates greater photoexcitation efficiency in the UV band (see Figure 1c). Although annealing temperature and photoexcitation efficiency do significantly affect the saturation photocurrent, we observe no such effects on the normalized relaxation in our samples. In principle, the structural properties and optical efficiency of films can correlate with the density/depth of surface traps; however, for our films, grown via a thermal oxidation technique, this is not observed.

### 4.3. Effect of Operating Temperature

**Figure 7.**
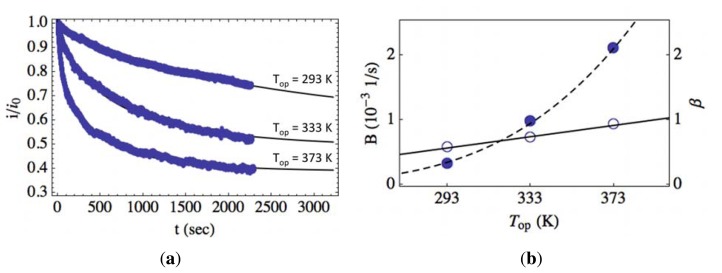
**(a)** Photocurrent transient relaxation with increasing temperature measured at 293, 333 and 373 K (solid circles) for intensity P = 0.2 mW/cm^2^. Experimental data is fit to Equation (11), with the best fits shown as the solid lines. For *T_op_* = (293, 333, 373) °C, *A* = (1.5, 1.5, 1.5) × 10^−2^*s*^−1^, *B* = (0.31, 0.97, 2.1) × 10^−3^*s*^−1^ and *β* = (0.57, 0.72, 0.92). (**b**) The fitting parameters, *B* (filled circles) and *β* (hollow circles), are plotted as a function of temperature and fit to an Arrhenius (dashed line) and linear (solid line) model, respectively.

The transient photoresponse of photodetectors having ZnO-based films annealed at 573 K and exposed to relatively low UV illumination intensity (P = 0.2 mW/cm^2^) are shown in Figure 7 for varying operating temperatures (*T_op_*). At an operating temperature of 293 K, transient relaxation was slower compared to detectors operating at 333 and 373 K, as shown in Figure 7a. The experimental data was fit to Equation (11), and the resulting fits are shown in Figure 7a as the solid lines. The fitting parameters were found to be *A* = (1.5, 1.5, 1.5) × 10^−2^*s*^−1^*B* = (0.31, 0.97, 2.1) × 10^−3^*s*^−1^ and *β* = (0.57, 0.72, 0.92) for *T_op_* = (293, 333, 373) K. Greater temperature results in a faster relaxation for the time period immediately after removal of illumination (*t* < 1,000 s); however, for longer times (*t* > 1,000 s), the total relaxation rate begins to slow with increasing temperature. It is seen that *β* controls what the literature defines as the “fast process,” and *B* contributes to the “slow process,” as is discussed in more detail in the next section [2,18].

Figure 7b shows the fitting parameters, *B* (filled circles) and *β* (hollow circles), plotted as a function of operating temperature. The fitting parameters as a function of temperature are fitted to an Arrhenius (dashed line) and linear (solid line) model for *B* and *β*, respectively. With increasing temperature, *β* is found to increase approximately linearly. This is consistent with the assumption that the density of photogenerated charge carriers is approximately proportional to the illumination intensity.

The coefficient, *B*, is fitted to an Arrhenius model, shown in Figure 7b as the dashed line. We expect such an Arrhenius relationship, as can be seen in the definition of *B* from Equation (6). Specifically, the values for *B* as a function of temperature were fit to the model, *a* exp [−*b*/(*kT*)], where the resulting value of *b* was found to correspond to a barrier height, Φ_0_ + *E_C_* − *E_F_*, of approximately 0.2 eV. This suggests that the assumed single surface state, *E_s_*, is located approximately 0.1 eV above the conduction band energy, *E_C_*, which is consistent with n-type ZnO having a native band bending of approximately 0.3 eV.

## 5. Discussion

Throughout the previous literature, a two-step photocurrent relaxation appears, which consists of an initial sharp drop in photoconductivity followed by a slow decline [17,19–21]. Previous literature offers suggestions to the nature of the energy transfer mechanisms that occur to give the transient relaxation curves their characteristic stretched-exponential shape. Typically, the photocurrent relaxation is explained via two physical processes: (1) a fast quasi-exponential decrease; and (2) a significantly slower decrease, contributing to the persistent photoconductivity. The primary fast drop in photoconductivity during photocurrent relaxation has been linked to the recombination of free electron-hole pairs coinciding with free excitation relaxation [19]. Reemts and Kittel attribute the slower portion of the decay, in which the photocurrent persistence is an order of magnitude longer than the fast, to a lattice relaxation process of surface states [17]. Li *et al.* discuss a model, where conduction-band electrons recombine with photogenerated holes at the surface after removal of illumination [36]. Surface band bending decreases during illumination, due to the migration of photogenerated holes to the surface. Immediately after turning off illumination, conduction-band electrons must overcome a relatively low energy barrier to recombine with photogenerated holes at the surface, contributing to fast initial relaxation. Covington *et al.* fit the normalized photocurrent transients to the linear combination of two exponentials with significant differences in time constants, corresponding to a “fast process” and a “slow process” for photocurrent relaxation [18].

The phenomenological model developed in Section 3.2 depends on two processes for charge transfer: (1) bulk-to-surface transfer of conduction band electrons through the potential energy barrier, due to surface band bending, which results in a decrease in photoconductivity; and (2) thermionic surface-to-bulk transfer of charge carriers through a fixed potential energy barrier, which results in increased photoconductivity. Initially, bulk-to-surface transfer dominates and, depending on the initial amount of band bending, can result in significant initial drops in photocurrent, typically ascribed as the fast process. As band bending increases, the rate of transfer is slowed and, eventually, must also compete with the surface-to-bulk transfer. This slowing of normalized photocurrent relaxation is typically ascribed as the slow process. In our model, these two processes are governed by the phenomenological parameters, *β* and *B*, respectively.

### 5.1. Persistent Photoconductivity and Surface Band Bending

**Figure 8.**
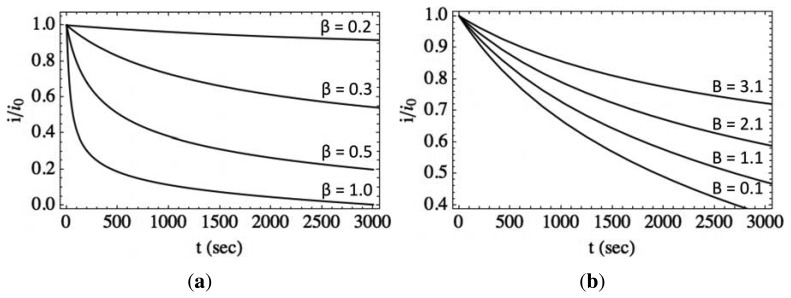
**(a)** Photocurrent transient relaxation model (Equation (11)) with varying values of *β* at room temperature and fixed values of *A* and *B*; (**b**) similarly, the model is shown with varying values of *B* with fixed values of *A* and *β*. The unit for values of *B* quoted in the figure is 10^−3^*s*^−1^.

Figure 8a shows the normalized photocurrent transient relaxation according to the model (Equation (11)) with varying values of *β* at room temperature and fixed values of *A* and *B*. With an increasing value of *β*, the initial decrease in normalized photocurrent becomes larger. This suggests that the ratio between the initial density of photogenerated electron/hole pairs and the available charged surface states governs the relaxation for short times. The ratio, *β*, also represents the percent of decrease in the native band bending due to illumination. For detectors undergoing a very little decrease in band bending due to illumination, the initial drop in normalized photocurrent is very small. The balance of surface-to-bulk and bulk-to-surface transfer of charge is moved only slightly from equilibrium and results in significant persistence in the photoconductivity within the first 1,000 s. Because the energy barrier for bulk-to-surface transfer remains relatively high, transfer of any photogenerated charge is slow.

For detectors undergoing a significant decrease in band bending due to illumination, the initial drop in normalized photocurrent is relatively high. In this case, the initial energy barrier for bulk-to-surface transfer of charge is low, resulting in initial fast transfer of photogenerated charge. Therefore, we find that *β*, or the illumination-induced decrease in band bending, governs the fast process discussed in the literature. This value will depend on both the density of photogenerated charge carriers and the number of surface traps, with the constant, *η*, determining the depth and nature of these surface traps. This is consistent with both the conceptual model proposed by Li *et al.* and previous reports, where time constants were found to be governed by the ambient environment and by the density and the depth of deep surface traps in ZnO, which affect the capture of holes and the subsequent hole emission and carrier recombination mechanisms of photocurrent decay [17,28,35,36].

Figure 8b shows the normalized photocurrent transient relaxation according to the model with varying values of *B* at room temperature and fixed values of *A* and *β*. The parameter, *B*, represents the surface-to-bulk contribution to the normalized photocurrent relaxation rate and includes information about the capture of charge at the surface. As *B* decreases, the rate of normalized photocurrent relaxation increases for *t* > 1, 000 s. This corresponds to either significant trapping of charge, reducing the ability of charge to transfer away from the surface, or a low probability of transfer, due to a relatively high energy barrier. In Figure 7a, we see the slope of *i*/*i_0_* as a function of time for *t* > 1, 000 s decreases with operating temperature. This is due to a decrease in surface charge trapping and/or an increase in transfer probability, due to the increase in thermal energy. Lower temperature results in lower probability of charge transfer over/through the barrier and could result in greater trapping by absorption of oxygen at the surface, for example, which leads to faster relaxation, due to the bulk-to-surface charge not competing with incoming surface-to-bulk transfer.

### 5.2. Limitations of the Model and Implications

Although the model presented in Section 3.2 fits the data well and provides a reasonable physical explanation for much of the observed phenomena associated with ZnO persistent photoconductivity, there are still several limitations to the model. Negative surface charge on *n*-type semiconductors has been attributed to disorder/defects at the interface and/or adsorption of species from the environment [37]. The exact nature of the initial negatively charged surface states is beyond the scope of the model presented in this work. Furthermore, a mechanism for charge trapping and/or scavenging due to interaction with the environment is not considered. Although the phenomenological factor, *η*, does provide information about the depth of surface traps, without direct measurement of the native band bending, the model has no predictive power in this respect. The parameters, Φ_0_ and *η*, could be different, while maintaining the fit, so long as the ratio between the two values remains constant. This allows only a range of values for Φ_0_ for the specified range of possible values of *η* [40].

What we can conclude is that the persistent photoconductivity is primarily a surface phenomenon, where the transfer of charge from the conduction band to photogenerated holes at the surface regulates the relaxation. This transfer is dependent on the surface band bending and the availability of initially charged surface and/or near surface states. We can also posit that the availability of negative surface charge is required for significant photoconductivity in ZnO detectors, since without electron/hole separation via surface band bending, recombination would proceed quickly in the form of photoluminescence, contributing little to increasing the conductivity. This is consistent with previous reports, where photoresponse has been attributed to the absorption and desorption of *O*_2_ at the surface, where, in dark, oxygen is absorbed by taking a free electron from the surface and, under illumination, photogenerated holes discharge the negatively charged oxygen ions, resulting in desorption of *O*_2_ from the surface.

## 6. Conclusions

In summary, we have presented a phenomenological model of the persistent photoconductivity observed in ZnO-based MSM planar photodetector devices based on time-resolved surface band bending. Surface band bending decreases during illumination, due to migration of photogenerated holes to the surface. Immediately after turning off illumination, conduction-band electrons must overcome a relatively low energy barrier to recombine with photogenerated holes at the surface; however, with increasing time, the adsorption of oxygen at the surface and/or electron trapping in the depletion region increases band bending, resulting in an increased bulk/surface energy barrier that slows transport of photogenerated electrons. Furthermore, charge transfer from the surface to the bulk over a fixed energy barrier contributes to conduction band charge carriers during long times, resulting in an increase in the persistence of the photoconductivity. Our complex rate Equation model for the transient relaxation based on these thermionic transitions numerically fits the empirical data well for both low and high intensity illumination and with variable temperature. Fitting parameters are found to be consistent with measured values in the literature. An understanding of the mechanism for persistent photoconductivity could lead to mitigation in future device applications.
